# Identifying complementary and alternative medicine recommendations for insomnia treatment and care: a systematic review and critical assessment of comprehensive clinical practice guidelines

**DOI:** 10.3389/fpubh.2023.1157419

**Published:** 2023-06-15

**Authors:** Fei-Yi Zhao, Peijie Xu, Gerard A. Kennedy, Russell Conduit, Wen-Jing Zhang, Yan-Mei Wang, Qiang-Qiang Fu, Zhen Zheng

**Affiliations:** ^1^School of Health and Biomedical Sciences, RMIT University, Bundoora, VIC, Australia; ^2^Department of Nursing, School of International Medical Technology, Shanghai Sanda University, Shanghai, China; ^3^Shanghai Municipal Hospital of Traditional Chinese Medicine, Shanghai University of Traditional Chinese Medicine, Shanghai, China; ^4^School of Computing Technologies, RMIT University, Melbourne, VIC, Australia; ^5^Institute of Health and Wellbeing, Federation University, Mount Helen, VIC, Australia; ^6^Institute for Breathing and Sleep, Austin Health, Heidelberg, VIC, Australia; ^7^Yangpu Hospital, School of Medicine, Tongji University, Shanghai, China

**Keywords:** complementary and alternative medicine, CAM, insomnia, sleep medicine, photo therapeutics, clinical practice guidelines, systematic review, quality assessment

## Abstract

**Background:**

There is a need for evidence-informed guidance on the use of complementary and alternative medicine (CAM) for insomnia because of its widespread utilization and a lack of guidance on the balance of benefits and harms. This systematic review aimed to identify and summarize the CAM recommendations associated with insomnia treatment and care from existing comprehensive clinical practice guidelines (CPGs). The quality of the eligible guidelines was appraised to assess the credibility of these recommendations.

**Methods:**

Formally published CPGs incorporating CAM recommendations for insomnia management were searched for in seven databases from their inception to January 2023. The NCCIH website and six websites of international guideline developing institutions were also retrieved. The methodological and reporting quality of each included guideline was appraised using the AGREE II instrument and RIGHT statement, respectively.

**Results:**

Seventeen eligible GCPs were included, and 14 were judged to be of moderate to high methodological and reporting quality. The reporting rate of eligible CPGs ranged from 42.9 to 97.1%. Twenty-two CAM modalities were implicated, involving nutritional or natural products, physical CAM, psychological CAM, homeopathy, aromatherapy, and mindful movements. Recommendations for these modalities were mostly unclear, unambiguous, uncertain, or conflicting. Logically explained graded recommendations supporting the CAM use in the treatment and/or care of insomnia were scarce, with bibliotherapy, Tai Chi, Yoga, and auriculotherapy positively recommended based on little and weak evidence. The only consensus was that four phytotherapeutics including valerian, chamomile, kava, and aromatherapy were not recommended for insomnia management because of risk profile and/or limited benefits.

**Conclusions:**

Existing guidelines are generally limited in providing clear, evidence-informed recommendations for the use of CAM therapies for insomnia management due to a lack of high-quality evidence and multidisciplinary consultation in CPG development. More well-designed studies to provide reliable clinical evidence are therefore urgently needed. Allowing the engagement of a range of interdisciplinary stakeholders in future updates of CPGs is also warranted.

**Systematic review registration:**

https://www.crd.york.ac.uk/prospero/display_record.php?RecordID=369155, identifier: CRD42022369155.

## 1. Background

Insomnia remains the most prevalent sleep complaint and is a major public health concern (1, 2). It is predominantly characterized by subjective perceptions of difficulty in initiating and/or maintaining sleep or experiencing non-refreshing and/or non-restorative sleep, often accompanied by reduced daytime performance and cognitive dysfunction (1, 3). Insomnia affects a considerable proportion of the general population globally as either a primary or a secondary comorbid condition (4). Up to one in five people suffer from insomnia or trouble sleeping based on data from the USA (5), Nordic countries (6) and South Korea (7). The COVID-19 pandemic is likely to have made insomnia more widespread. A systematic review including 98 studies with 193,889 Chinese participants suggested that 39.1% presented insomnia during the COVID-19 pandemic and insomnia symptoms did not improve despite control of the disease (8). Researchers propose that insomnia associated with suffering COVID-19 appears to persist over time (8). Insomnia suffers have reduced work productivity, and higher rates of absenteeism, accidents, and hospitalization, impaired memory function, complaints of daytime fatigue, and lower quality of life (3, 9). The aggregate total of direct and indirect costs of insomnia has been estimated to exceed 100 billion US dollars per annum (9). Furthermore, Insomnia is associated with a heightened risk of hypertension (10), cardiac disease (10, 11), type 2 diabetes (11, 12), obesity (11), depression (3, 10), and suicide (2).

Cognitive-behavioral therapy for insomnia (CBTi) is considered the frontline insomnia treatment with well-established efficacy (11, 13). Yet, delivery of CBTi has been limited by the scarcity of trained therapists (13). Hypnotic medications, the second line of treatment, are therefore widely prescribed by clinicians (11). However, growing clinical concerns regarding sedatives/hypnotics with respect to potential abuse, adverse events, dependence, and withdrawal issues have been reported (14, 15). Whilst CBTi and pharmacotherapy with mainly hypnotics remain the mainstays of conventional treatment, interest in the utilization of complementary and alternative (CAM) therapies for managing insomnia has emerged (16). CAM is defined as an array of diverse medical- and health- related systems, practices, and products that are not presently considered part of biomedicine-oriented mainstream or conventional healthcare systems (17, 18). According to the *National Center for Complementary and Integrative Health* (NCCIH, updated on March 2023), CAM modalities are generally classified into five categories depending on their primary therapeutic input: nutritional (e.g., probiotics, dietary supplements, etc.), physical (e.g., heat/cold therapies, massage), psychological (e.g., spiritual practice, mindfulness), combinations such as psychological and physical (e.g., Tai chi, yoga) or psychological and nutritional (e.g., mindful eating), and other complementary health approaches (19). Insomnia or trouble sleeping has been well-documented as one of the top five medical complaints for which CAM is most commonly used (20). Analyses from the 2002 USA National Health Interview Survey data showed that amongst the 17.4% of 93,386 adults regularly reporting insomnia or trouble sleeping, 4.5% utilized CAM to improve their condition (21). This result can be extrapolated to over 1.6 million non-institutionalized USA civilians (21). An online cross-sectional survey involving 2019 Australians revealed that of 13% respondents living with sleep disorders, 63.8% used complementary medicine (22). Using CAM is more popular among some Asian populations. Yeung et al. reported that CAM was utilized more often than orthodox medical therapies for trouble sleeping in the general population in HongKong (20).

Despite the increased demand and prevalence of CAM use, conventional healthcare practitioners receive little to no specialized and systematic education or training in respect to CAM (23, 24). This lack of education/training can result in poor communication between practitioners and their customers when discussing CAM use for insomnia management, which further hampers treatment efficacy and other clinical outcomes (23, 25). Healthcare service providers use evidence-based clinical practice guidelines (CPGs) to inform their decision making in clinical practice, particularly in fields where their knowledge and expertise may be lacking (23, 26). The CPGs containing CAM components can also assist practitioners inform patients on the pragmatic and judicious use of CAM therapies, including advising against using therapy where there is clear evidence of non-efficacy and/or harmful side-effects, or recommending therapy use when benefits may outweigh the risks (27, 28). Based on the existing literature, several CAM modalities such as valerian (29), meditative movement (30), hypnotherapy (31), acupuncture (32), etc. have shown potentials in insomnia amelioration. Have these modalities been incorporated into and recommended by existing CPGs for clinical practitioners (particularly those Western medical clinicians/registered nurses without a CAM background) as potential options for insomnia treatment and/or care? What is the strength of these CAM recommendations? Are there CAM modalities perceived as ineffective or even harmful and thus strongly discouraged by the existing CPGs for use? Bridging these knowledge gaps is of significant clinical relevance and prompted us to conduct the current systematic review.

## 2. Materials and methods

### 2.1. Registration and eligibility criteria

The approaches employed for the present systematic review were consistent with the guidelines detailed on *Preferred Reporting Items for Systematic Reviews and Meta-Analyses (PRISMA) 2020 Statement* checklist (33). The protocol for this systematic review was registered with PROSPERO (Identifier: CRD42022369155). Only formally published sleep-related CPGs containing CAM recommendations for treatment and/or care of adult insomnia were included in the current review. The form of CAM therapy and type of insomnia [primary insomnia or insomnia associated with or secondary to other medical conditions (e.g., insomnia in cancer survivors, perimenopausal insomnia, etc.)] were not limited. The specific modality and attribute of various CAM therapies could refer to the classification updated by the NCCIH (19). The publication date of the CPGs was not limited, while the language was restricted to English and/or Chinese. As clarified in the “*Background*” section, this review was primarily interested in capturing the CAM recommendations in CPGs whose users were Western medical practitioners. Therefore, only comprehensive CPGs were considered. Those specialized CAM CPGs (i.e., homeopathic, herbalism, acupuncture, or Ayurveda CPGs) were not included. The CPGs were also excluded if they (1) did not include any CAM recommendation associated with insomnia in adults; (2) were earlier versions of CPGs with an available updated version; and (3) did not clearly describe the systems or methods used for grading the evidence and recommendations.

### 2.2. Data sources and searches

Following consultation with a professional librarian with a health science background who assisted in development of the overall search strategy, we used filters to reliably identify relevant CPGs, and undertook a comprehensive search of three English electronic databases and four Chinese electronic databases—AMED: Allied and Complementary Medicine Database, EMBASE (via OVID), MEDLINE (via PubMed), Chongqing VIP database (CQVIP), Wanfang database, China National Knowledge Infrastructure (CNKI), and China biomedical literature service system (SinoMed)—from their launch through to January 2023. The search strategies (Appendix 1) included indexed headings and keywords that reflect terms commonly used in the literature to refer to insomnia and guidelines. These terms used for searching were developed based on the librarian's suggestions and the search strategies in two published systematic reviews with a relevant theme (23, 34). To ensure literature saturation, searches were also performed by a manual retrieval in a single list of CAM CPGs provided by NCCIH website (https://www.nccih.nih.gov/health/providers/clinicalpractice), as well as six websites of international guideline developing institutions, namely Guidelines International Network (GIN, https://g-i-n.net/), British Columbia guideline (BC Guidelines, http://www.bcguidelines.ca/alphabetica), National Guideline Clearinghouse (NGC, https://www.ahrq.gov/gam/index.html), Scottish Intercollegiate Guidelines Network (SIGN, https://www.sign.ac.uk/), Canadian Medical Association: Clinical Practice Guideline (CMA-CPG Infobase, https://joulecma.ca/), and National Institute for Health and Clinical Excellence (NICE, https://www.nice.org.uk/).

### 2.3. Selection of CPGs and data extraction

Two screeners (PJ-X and FY-Z) independently screened the titles and abstracts for eligibility by using the Rayyan (35). The full-text was then acquired and cross-checked for eligibility (WJ-Z and QQ-F) by using Microsoft Office Excel (Version 2021). Two standardized and predetermined data forms were employed to extract the following information from each CPG: identification/demographic information [first author, year of publication, country of first author, and primary developer/publishing entity (i.e., professional associations or societies, research institutions, government departments)], design basis of the guideline [evidence-based or consensus-based, target population (patients with primary insomnia or insomnia secondary to other diseases), version (original or updated)], retrieval-related information (search year covered, and databases and search strategy used), funding received (if available), the criteria used for evaluating the level of evidence and the system used for grading the strength of recommendations, as well as the modalities of the CAM included in each CPG. CAM recommendation levels in each CPG were also extracted and are presented as a figure.

### 2.4. Quality appraisal of CPGs

#### 2.4.1. Methodological quality appraisal

The methodological quality of the included CPGs was critically assessed by four independent appraisers (QQ-F, WJ-Z, YM-W, and FY-Z) using the Appraisal of Guidelines Research and Evaluation (2nd version; AGREE II) instrument (36). Prior to the appraisal practice, these four appraisers were trained and pretested the use of AGREE II instrument to ensure they had a thorough understanding of each item of this instrument and to increase internal agreement. The AGREE II instrument comprises 23 appraisal criteria (items), rated on a 7-point scale (1 = strongly disagree; 7 = strongly agree) and organized within six domains, namely scope and purpose, stakeholder involvement, rigor of development, clarity of presentation, applicability, and editorial independence (37). For each domain, the scores are summed up and calculated using the following formula: [(score obtained – minimum possible score)/(maximum possible score – minimum possible score)] × 100. The possible standardized scores range from 0% (the minimum) to 100% (the maximum). A previous study suggested that to reflect the overall score of a CPG, the global score could be obtained by calculating the sum of the six domain scores and dividing by 600%, with a global score ranging from 0 to 100% (38). AGREE II instrument does not define a standard association between the global score and guideline quality, while a previous study suggested a CPG with global score of < 50% as low-quality CPG, 50–70% as adequate (moderate)-quality CPG, and > 70% as good-quality CPG (38).

#### 2.4.2. Reporting quality appraisal

The Reporting Items for practice Guidelines in Healthcare (RIGHT) checklist was adopted to evaluate the reporting quality of included CPGs by two independent appraisers (WJ-Z and YM-W) (39). The RIGHT checklist comprises 35 appraisal criteria (items) grouped into seven domains: basic information, background, evidence, recommendations, review and quality assurance, funding, declaration and management of interests, as well as information. Each item was rated as “Yes” (guideline reported majority information), “No” (relevant information on the item was not reported) and “N/A” (not applicable, the item did not need to be evaluated due to certain features of the guideline). Any discrepancy between two quality appraisers was assisted in resolving with the assists and thorough discussion with a third evaluator (QQ-F). The number of reported items of each CPG was documented.

### 2.5. Data synthesis

We calculated the mean score and the standard deviation of all included CPGs in each domain of AGREE II instrument, which contributes to an overall understanding of the average level of quality of CPGs in each dimension. Likewise, we calculated the reporting rate for all included CPGs in each item of the RIGHT checklist to understand in which dimensions CPGs usually report completely/incompletely. A stacked polar chart and a clustered bar chart were adopted to visualize the assessment results from the AGREE II instrument and the RIGHT checklist, respectively. The Origin Pro (Version 2022), Microsoft Office Excel (Version 2021), Microsoft Office PowerPoint (Version 2021) were used to create these two figures. Given the AGREE II instrument was rated by four assessors separately, we introduced the Intraclass Correlation Coefficients (ICCs) with 95% confidence intervals to measure the agreement across all assessors for each item of AGREE II and thus appraise inter-rater reliability. Such data can also reflect the credibility of the assessment results of AGREE II from the side. The ICCs statistics were run using SPSS software (Version 26.0) with the reliability analysis module. The strength of agreement for ICC point estimates was considered poor (0.01 – 0.20), fair (0.21 – 0.40), moderate (0.41 – 0.60), good (0.61 – 0.80), or excellent (0.81 – 1.00) (40).

In addition, we built a bubble plot using Origin Pro to show the overall quality of each included CPG comprehensively, with the Y-axis denoting the global scores of the AGREE II and X-axis denoting the average reporting rate of the RIGHT checklist. Accordingly, all included CPGs were divided into three clusters: high-quality CPG (80 ≤ *X* value and 70 ≤ *Y* value), moderate-quality CPG (55 ≤ *X* value < 80 and 50 ≤ *Y* value < 70), or low-quality CPG (*X* value < 55 and *Y* value < 50). The three colored spheres, namely green (high quality), yellow (moderate quality) and red (low quality) were adopted to distinguish and visualize the overall quality of each CPG. Based on the findings in this bubble plot, we summarized and analyzed the reliability and applicability of CAM recommendations derived from the CPGs. Neither the AGREE II instrument nor the RIGHT checklist defined the link between the scores and recommended strengths, while we here suggest the high-, moderate- and low-quality CPGs visualized in the bubble plot as “recommended,” “recommended with modifications,” and “not recommended.”

## 3. Results analysis

### 3.1. CPGs selection

**Figure 1:**
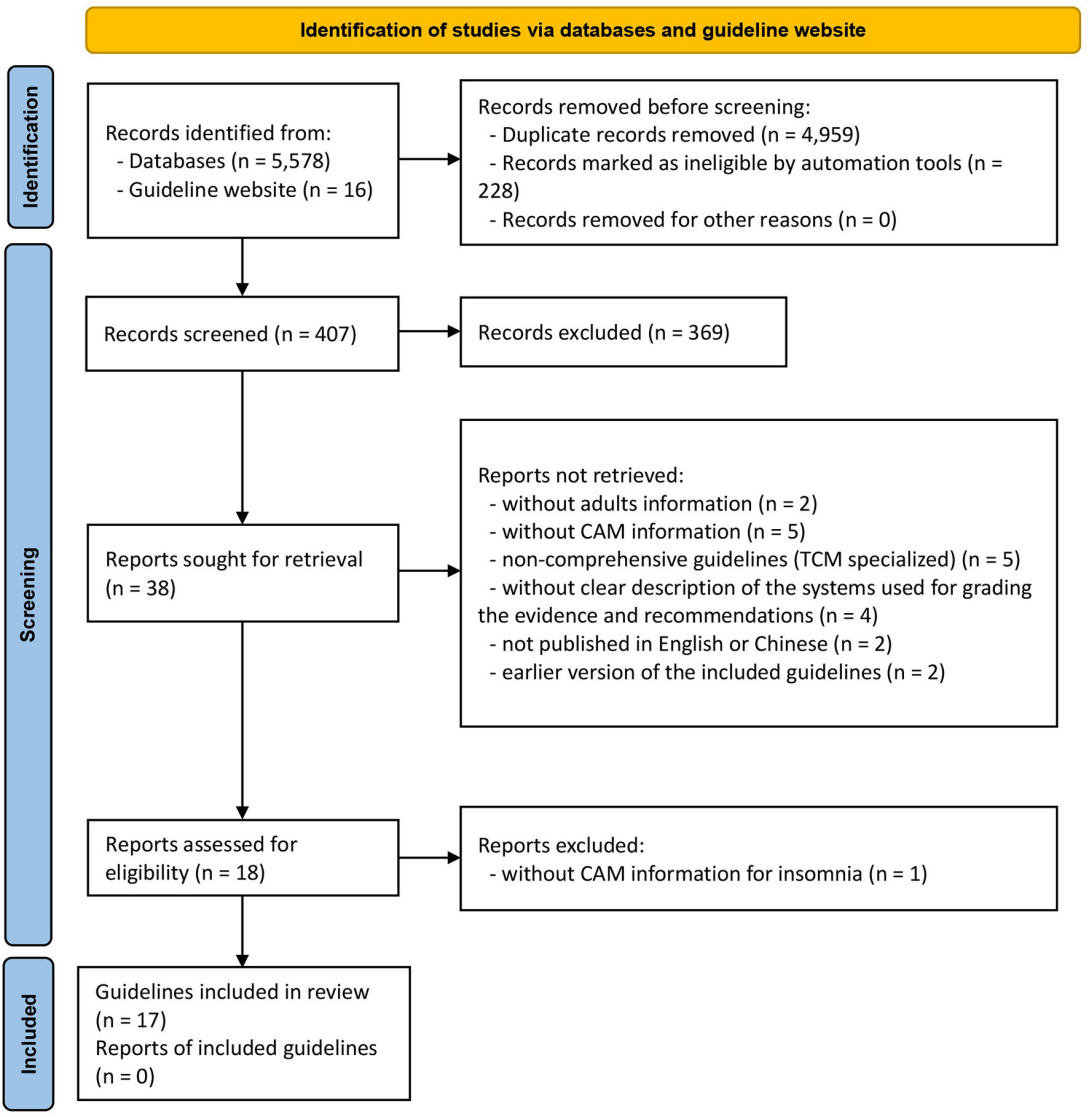
Flow diagram of the study selection process.

A total of 5,594 works were identified using our search strategy in the initial search. After removal of the duplicates and literatures with unrelated titles/abstract in the preliminary screening process, 38 CPGs were found. Followed by a further careful full-text screening, 21 CPGs were excluded, and the remaining 17 CPGs eventually met the predefined criteria (Figure 1). Amongst them, two CPGs (41, 42) were published in Chinese and the remaining CPGs were published in English. The discarded 21 CPGs with detailed justifications for exclusion are shown in Appendix 2.

### 3.2. CPGs characteristics

The features of the 17 included CPGs are extracted and summarized in Table 1. Eligible CPGs were published from 2003 to 2021, in the United States (*n* = 9), China (*n* = 2), Spain (*n* = 1), Korea (*n* = 1), Canada (*n* = 1), Philippines (*n* = 1), Brazil (*n* = 1), or Germany (*n* = 1). Five CPGs (29.4%) (42–46) were the updated version.

**Table 1:** Characteristics of the eligible clinical practice guidelines.

**References**	**Evidence-based (EB), or consensus-based (CB)**	**Population**	**Diagnosis**	**Country**	**Primary developer/ publishing entity**	**Version**	**Systematical search included**	**Databases**	**Search strategies**	**Search year**	**Funding**	**CAM modalities included**
Artiach et al. (54)	Both EB and CB	General	DSM-IV-TR	Spain	NHSIC	Original	Yes	Medline, Embase, PsycINFO, CINAHL, Cochrane Plus, DARE, HTA, Clinical Evidence, INAHTA, NHS EED, CINDOC in Spanish, English and French	Yes	NR	Carlos III, HTAULEA	Melatonin, valerian, acupuncture, and bibliotherapy
Baker et al. (48)	EB	Middle-aged to older adults (≥45)	NR	USA	SN-UTA	Original	Yes	Medline, PubMed, CINAHL Plus, Cochrane, PsycINFO, and PsycARTICLES	Yes	2000–2014	Family Nurse Practitioner Program (SN-UTA)	Melatonin, valerian, Tai Chi, acupuncture, acupressure, light therapy, massage, yoga, and tart cherry juice
Bloom et al. (49)	EB	Elderly (≥65)	Have difficulty falling asleep/staying asleep ≥ 1 month + causes impairment in daytime functioning	USA	Third conference of ILC	Original	Yes	PubMed, CDSR, NGC, CRD/HTAD	NR	NR	NR	Tai Chi, acupressure
Choi et al. (55)	Both EB and CB	General	ICSD and DSM-V	Korea	KNA	Original	Yes	PubMed, EMBASE + various medical guideline website (e.g., NGC, NICE, GIN, etc.)	NR	2015–2020	KNA	Valerian and melatonin
Denlinger et al. (43)	Both EB and CB	Cancer survivors	[Have difficulty in falling asleep and/or maintaining sleep ≥ 3 times per week] ≥ 4 weeks, accompanied by distress	USA	NCCN	Updated	Yes	NR	NR	NR	NCCN	Valerian and melatonin
Devlin et al. (44)	Both EB and CB	ICU patients	NR	USA	ACCM	Updated	Yes	PubMed, EMBASE, Cochrane, CINAHL, and WOS	Yes	1990–Oct 2015	NIA, NHLBI, AZP	Melatonin, music therapy, aromatherapy, and acupressure
Edinger et al. (45)	Both EB and CB	General	ICSD-3 and DSM-V	USA	AASM	Updated	Yes	PubMed, PsycINFO	Yes	- Jan 2017	AASM	Biofeedback and mindfulness
Han et al. (41)	Both EB and CB	General	ICSD-3	China	CSRS	Original	Yes	PubMed, EMBASE, Cochrane, CNKI, and WanFang	NR	July 1999–Dec 2015	NR	Melatonin, light therapy, biofeedback, music therapy, CHM, and acupuncture
Howell et al. (47)	Both EB and CB	Cancer survivors	ICSD and DSM-IV	Canada	CAPO and CPAC	Original	Yes	MEDLINE, EMBASE, PsycINFO, HealthStar, Cochrane, CPACICG, GIN, AASM, NGC, NICE, SIGN, NCCN, and PGOs	Yes	2004–June 2012	Health Canada	Melatonin, valerian, massage, yoga, aromatherapy, music therapy, acupuncture, and homeopathy
Leopando et al. (50)	EB	General	DSM-IV	Philippines	M-UP	Original	Yes	MEDLINE, OVID, and internet resources	NR	1966–2002	NR	Melatonin
Mysliwiec et al. (51)	EB	VA and DoD patients	DSM-IV	USA	V/DEBP	Original	Yes	PubMed, MEDLINE, CDSR, EMBASE (Excerpta Medica), PsycINFO, and DARE	Yes	Jan 2008–May 2018	DCI-ADATP	Mindfulness meditation, auriculotherapy, acupuncture, Tai Chi, yoga, Qigong, valerian, chamomile, kava, and melatonin
Pinto et al. (52)	EB	General	ICSD-2 and DSM-IV	Brazil	BSA	Original	Yes	NR	NR	NR	NR	Valerian
Qaseem et al. (53)	EB	General	ICSD-3 and DSM-V	USA	ACP	Original	Yes	MEDLINE, EMBASE, CENTRAL, PsycINFO bibliographic databases	NR	2004–Sept 2015	ACP operating budget	Melatonin, acupuncture, and CHM
Riemann et al. (56)	Both EB and CB	General	ICSD-3 and ICD-10	Germany	ESRS	Original	Yes	PubMed, Cochrane, journal (Sleep Medicine Reviews)	Yes	Jan 1966–June 2016	ESRS	Melatonin, valerian, chamomile, kava, hops, melissa, passiflora, acupuncture, moxibustion, aromatherapy, foot reflexology, homeopathy, yoga, light therapy, and mindfulness
Sateia et al. (46)	Both EB and CB	General	ICSD-3	USA	AASM	Updated	yes	PubMed	yes	- Jan 25^th^, 2016	AASM	valerian, melatonin
Schutte-Rodin et al. (57)	Both EB and CB	General	ICSD-2	USA	AASM	Original	Yes	MEDLINE	NR	1999–Oct 2006	No industry support	Valerian, melatonin, and biofeedback
Zhang et al. (42)	Both EB and CB	General	ICSD-3 and DSM-V	China	CMA	Updated	Yes	NR	NR	Jan 2012–Aug 2017	NR	Melatonin, light therapy, biofeedback, aromatherapy, massage, homeopathy, and CHM

NR, no reports; AASM, American Academy of Sleep Medicine; ACCM, American College of Critical Care Medicine; ACP, American College of Physicians; AZP, AstraZeneca Pharmaceuticals; BSA, Brazilian Sleep Association; Carlos III, Carlos III Health Institute; CAPO, Canadian Association of Psychosocial Oncology; CDSR, Cochrane Database of Systematic Reviews; CHM, Chinese herbal medicine; CMA, Chinese Medical Association; CNKI, China National Knowledge Infrastructure; CPAC, Canadian Partnership Against Cancer; Cochrane, Cochrane Library; CPACICG, Canadian Partnership Against Cancer's Inventory of Cancer Guidelines; CRD/HTAD, Center for Reviews and Dissemination/Health Technology Assessment databases; CSRS, Chinese Sleep Research Society; DCI-ADATP, Durham Center of Innovation to Accelerate Discovery and Practice Transformation (ADAPT) at the Durham VA Healthcare System; DSM-IV, Diagnostic and Statistical Manual of Mental Disorders (Fourth Edition); DSM-IV-TR, Diagnostic and Statistical Manual of Mental Disorders (Fourth Edition-Text Reversion); DSM-V, Diagnostic and Statistical Manual of Mental Disorders (Fifth Edition); DoD, US Department of Defense; DARE, Database of Abstracts of Reviews of Effects; ESRS, European Sleep Research Society; GIN, Guidelines International Network; HTAULEA, Health Technology Assessment Unit of the Laín Entralgo Agency; ICSD-2, International Classification of Sleep Disorders (Second Edition); ICSD-3, International Classification of Sleep Disorders (Third Edition); ILC, International Longevity Center; KNA, Korean Neuropsychiatric Association; M-UP, College of the Medicine-University of the Philippines; NCCN, National Comprehensive Cancer Network; NIA, National Institute of Aging; NHLBI, National Heart, Lung and Blood Institute; NGC, National Guideline Clearinghouse; NHSIC, National Health System Interterritorial Council; NICE, National institute for health and care excellence; PGOs, provincial guideline organizations; SIGN, Scottish Intercollegiate Guidelines Network; SN-UTA, School of Nursing, The University of Texas at Austin; VA, US Department of Veterans Affairs; V/DEBP, VA/DoD Evidence-Based Practice Work Group; WOS, Web of Science.

Whilst all CPGs focused on adult insomnia, target populations varied across these guidelines. Classified by the type of insomnia, two CPGs were designed for insomnia in cancer survivors (43, 47), one CPG was designed for insomnia in intensive care unit (ICU) patients (44), and the remaining CPGs were not limited to any particular group. Classified by the sociodemographic characteristics, one CPG was designed for middle-aged to older adults (≥45 years old) (48), one CPG was designed for older adults (≥65 years old) (49), and the remaining CPGs did not limit the age.

In 13 included CPGs, the diagnostic criteria for insomnia were referenced from recognized diagnostic manuals (e.g., ICD-10, DSM-IV, DSM-V, and ICSD-3). Of the remaining four CPGs, the diagnostic criteria for insomnia in two CPGs were defined by the consensus of the experts involved in the development of such CPG (43, 49); and the other two CPGs did not provide detailed information on the diagnostic criteria for insomnia (44, 48).

Six of the 17 CPGs were developed based on evidence only (48–53), and the remaining CPGs were developed based on both evidence and expert consensus. All CPGs were evidence-based with systematic literature searches. However, three CPGs (42, 43, 52) did not describe the databases that were used for retrieval; nine CPGs did not detail the specific search strategies.

The 17 included CPGs involved a total of 10 grading systems adopted to quantify the level of evidence and the strength of recommendation. Of these, seven CPGs used the GRADE system; three CPGs used the original or modified *American Academy of Sleep Medicine (AASM)* system; and the remaining seven CPGs used *Scottish Intercollegiate Guidelines Network (SIGN)* system, *National Comprehensive Cancer Network (NCCN)* system, *American Geriatrics Society (AGS) Panel* system, *Canadian Medical Association* system, *National Guideline Clearinghouse (NGC)* system, *Chinese Medical Association (CMA)* system, and *United States Preventive Services Taskforce (USPSTF) Ratings* system, respectively (Table 2).

**Table 2:** Grading systems adopted in the included clinical practice guidelines.

**Grading system**	**Codes of evidence and recommendation**		**Number of CPGs**	**CPGs**
	**Levels of evidence**	**Strengths of recommendation**		
GRADE	High, Moderate, Low, and Very low	Strong, Weak	7	(44–46, 51, 53, 55, 56)
AASM	1, 2, 3	Standard, Guideline, Option, Consensus	2	(41, 57)
Modified AASM	I, II, III, IV, V	Standard, Guideline, Option, not recommended	1	(52)
SIGN	1 ++, 1 +, 1 -, 2 ++, 2 +, 2 -, 3, 4	A, B, C, D (+ √^2^, Q)	1	(54)
NCCN	Poor, Low, Average, Good, and High	1, 2A, 2B, 3	1	(43)
AGS Panel	I, II, III	A, B, C, D, E	1	(49)
Canadian Medical Association system^a^	Good, Fair, Poor	A, B, C, D, E	1	(50)
NGC	N/A	A, B, C	1	(47)
CMA^b^	1, 2, 3, 4	I, II, III, IV	1	(42)
USPSTF ratings	High, Moderate, Low	A, B, C, D, I	1	(48)

^a^Developed by the Canadian Task Force on Preventive Health Care of the Canadian Medical Association.

^b^Developed based on the Oxford Center for Evidence-Based Medicine (OCEBM) Levels of Evidence.

AASM, American Academy of Sleep Medicine; AGS, American Geriatrics Society; CMA, Chinese Medical Association; GRADE, Grading of Recommendations Assessment, Development and Evaluation; NCCN, National Comprehensive Cancer Network; NGC, National Guideline Clearinghouse; SIGN, Scottish Intercollegiate Guidelines Network; USPSTF, United States Preventive Services Taskforce.

### 3.3. Quality of CPGs

#### 3.3.1. Methodological quality of CPGs

There was good to excellent inter-rater reliability (IRR) across the four appraisers in methodological quality assessment, with the overall ICCs statistics varying from 0.73 [95% CI (0.54–0.88), *p* < 0.01] to 0.90 [95% CI (0.81–0.96), *p* < 0.01] (Appendix 3).

**Figure 2:**
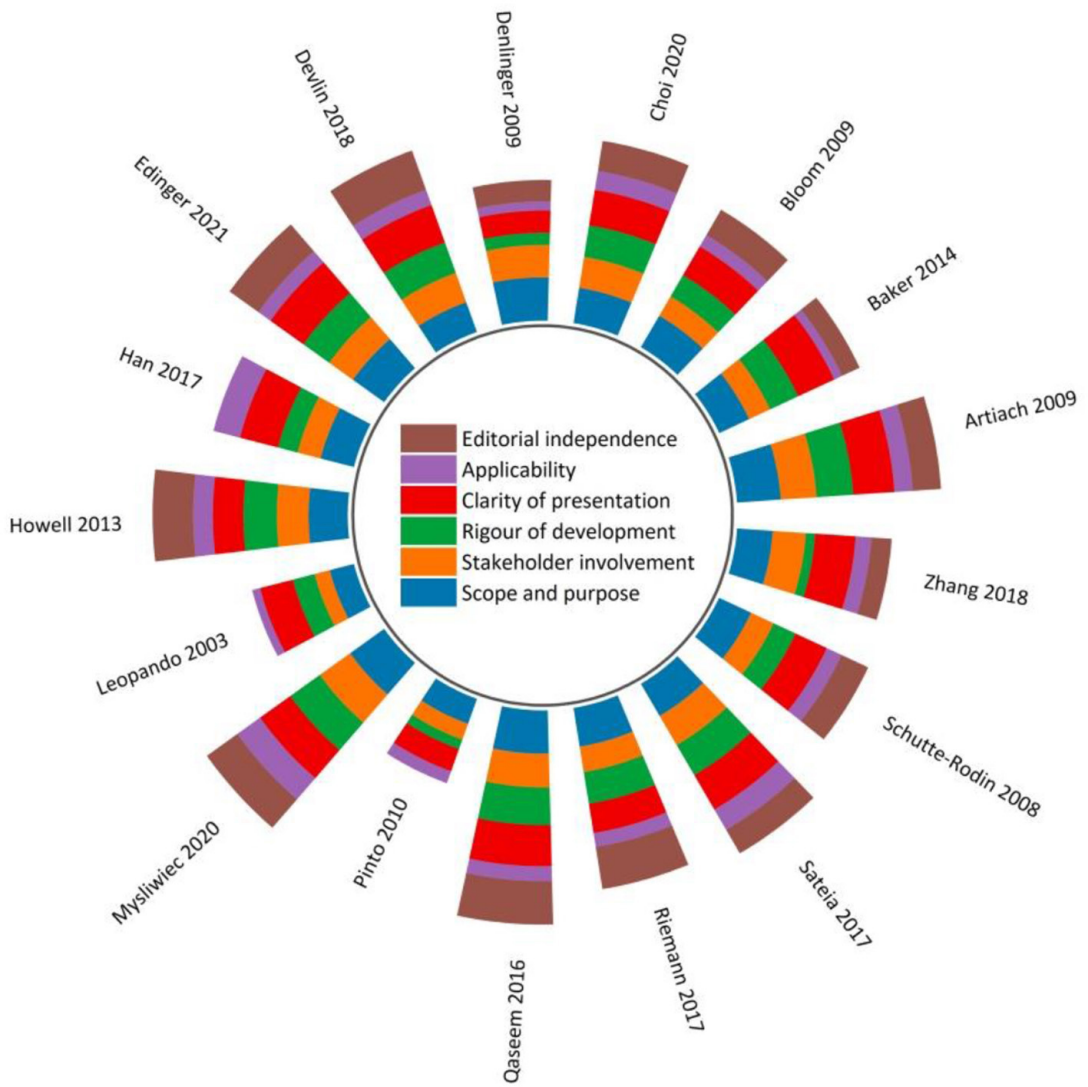
Global AGREE II scores by domain across 17 clinical practice guidelines.

Figure 2 and Appendix 4 display the sum of the AGREE II scores of each eligible CPG. Three CPGs (17.6%) (51, 53, 54) were rated as high-quality, four (23.5%) (41, 43, 50, 52) were low-quality, and the remaining were moderate-quality. Among the four low-quality guidelines, three (41, 50, 52) were scored at 0% for the “Editorial independence” domain due to a lack of transparent information with respect to either the competing interests of CPG development panel members and/or the influence of the funding body on the CPG recommendations. Whilst competing interests were addressed and reported in the remaining CPGs, most of them failed to report the methods used to seek competing interests, and/or the types of competing interests considered.

With regards to scaled domain percentages of CPGs, “Scope and purpose” domain achieved the highest average score (75.0 ± 11.1%), suggesting that the overall objectives, health questions, and population for whom the CPG was meant to apply were well-defined except for the two included CPGs that scored < 60% (50, 52). This was followed by the “Clarity of presentation” domain (72.9 ± 12.3%), which required the recommendations to be specific and unambiguous, key recommendations to be easily accessible, and different options for various conditions to be presented conspicuously.

The lowest average score appeared in the “Applicability” domain (33.8 ± 11.9%). Without detailed descriptions of facilitators and barriers to the CPG utilization, direct advice and/or tools supporting the implementation of the recommendations, and/or information concerning monitoring and/or auditing criteria, fourteen CPGs (82.4%) received lower scores in this domain compared to other domains. Only three CPGs relatively adequately addressed the resource implications of implementing the recommendations (41, 47, 51).

The “Stakeholder involvement” (58.2 ± 13.2%) and the “Rigor of development (55.6 ± 19.3%)” were two domains with scores slightly below the average scores of all six domains (58.3 ± 12.4%). In “Stakeholder involvement” domain, target users in most CPGs were typically well-defined. These guidelines usually provided thorough details in reference to the characteristics of the guideline development panel members, including their names, professions, and institutional affiliations. However, few CPGs tried to seek the views and preferences of the target population through reasonable strategies and/or detailed this information. Because of overall methodological rigor, two CPGs (51, 54) scored relatively high in the “Rigor of development” domain. In this domain, most CPGs lost scores in item 13 (external review of the CPG by experts prior to its publication) and item 14 (a procedure for updating the guideline is provided).

#### 3.3.2. Reporting quality of CPGs

**Figure 3:**
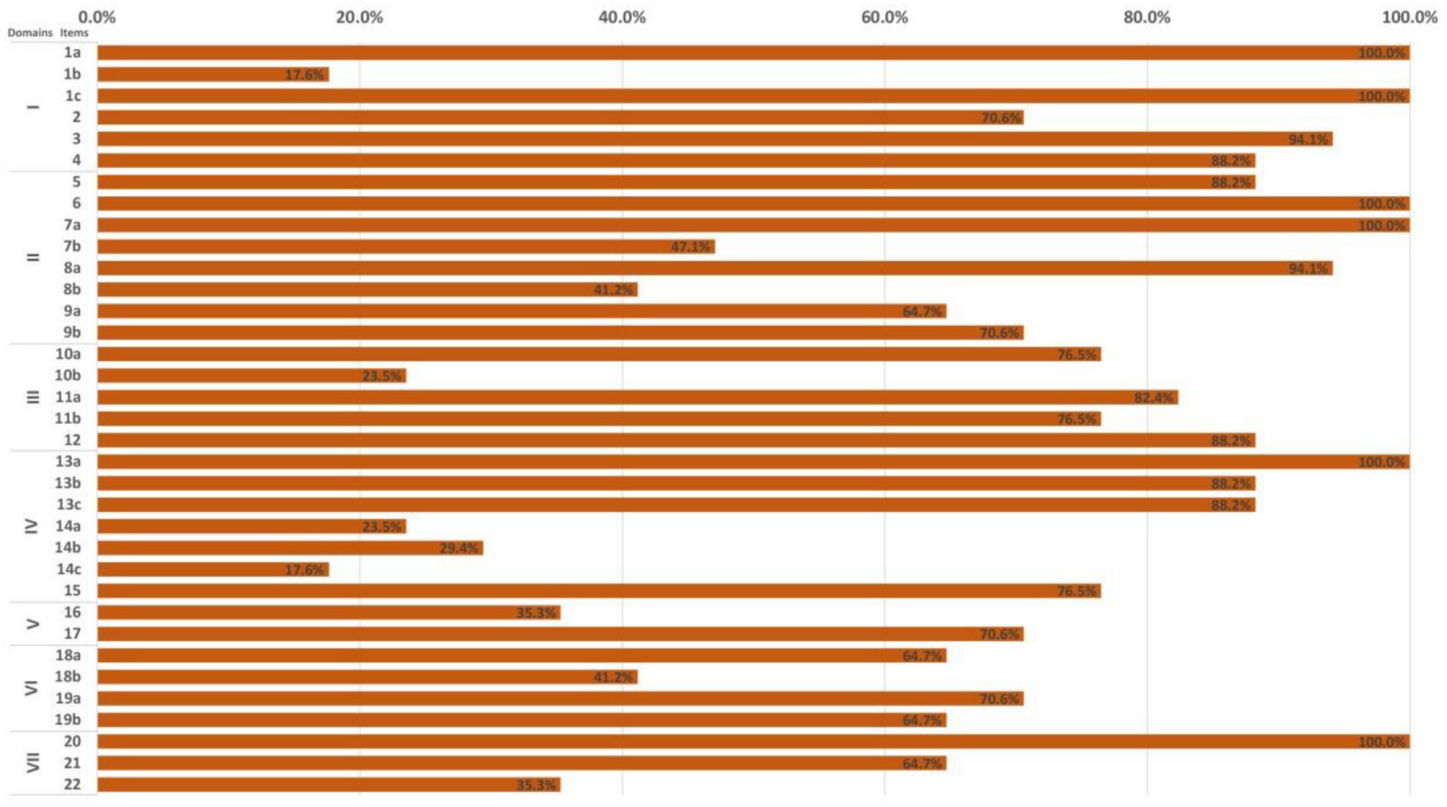
Overall reporting rate of by RIGHT items across 17 clinical practice guidelines.

In the light of the RIGHT checklist, the overall reporting rate of the 17 included CPGs ranged from 42.9 to 97.1%. Nearly half of the CPGs (*n* = 8, 47.1%) had an overall reporting rate higher than 75.0% (Figure 3 and Appendix 5).

Of the seven domains, the three with the highest reporting rate were, in descending order, “Basic information” (78.4%), “Background” (75.7%), and “Evidence” (69.4%) domain. The “Review and quality assurance” domain showed the lowest reporting rate (53.0%). Five items had significant reporting deficiencies (reporting rate ≤ 30%), namely 1b (year of publication; 17.6%), 10b (selection and sequencing of outcomes; 23.5%), 14a (values/preferences of the target population; 23.5%), 14b (cost and resource implications; 29.4%), 14c (other factors associated with the recommendations formulation; 17.6%). Six items (i.e., 1a, 1c, 6, 7a, 13a, and 20) were completely reported in all reviewed CPGs (Figure 3 and Appendix 5).

#### 3.3.3. Overall quality of CPGs

**Figure 4:**
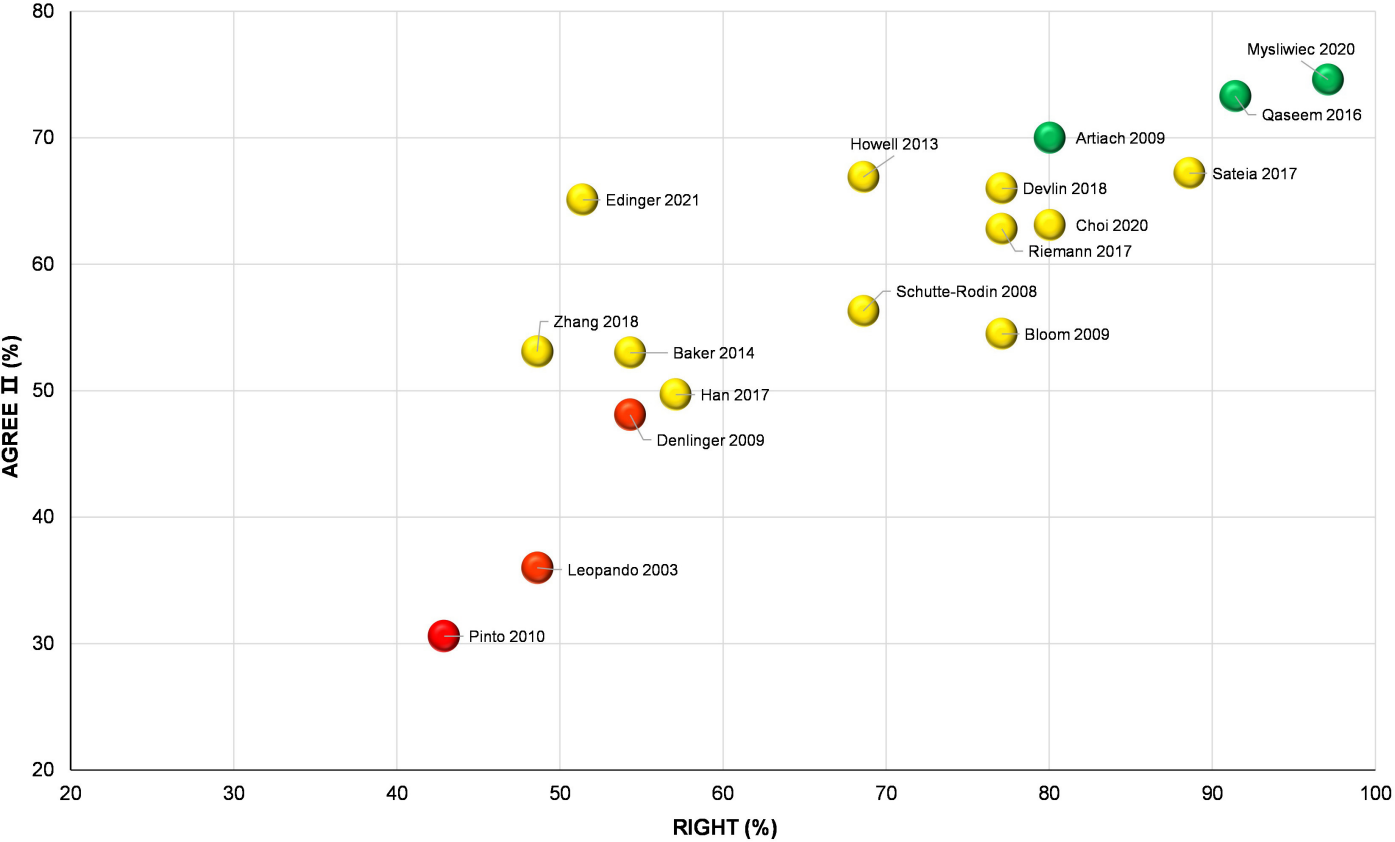
Grading and analysis of overall quality across 17 clinical practice guidelines.

In accordance with the bubble plot, three CPGs (51, 53, 54) were identified as high-quality guidelines and could be recommended, three guidelines (43, 50, 52) were identified as low-quality guidelines and should not to be recommended, and the remaining 11 CPGs were identified as moderate-quality guidelines and required modification before being recommended (Figure 4).

### 3.4. Recommendations of CAM

**Figure 5:**
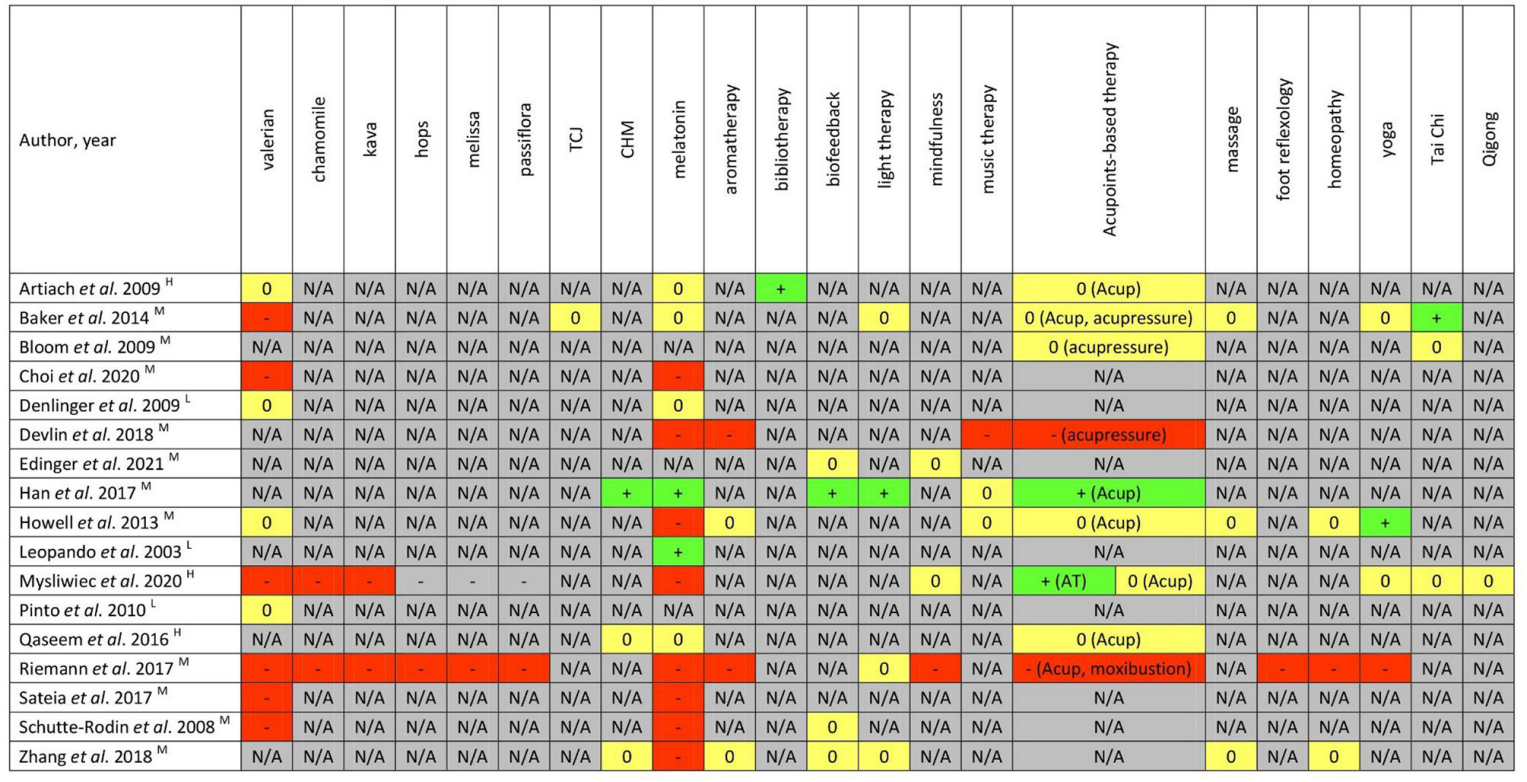
Summary of CAM recommendations in each clinical practice guideline. +/green = recommendations supporting the therapy use; -/red = recommendations against the therapy use; 0/yellow = recommendations unclear, uncertain, conflicting, or “neither for nor against”; N/A/gray = no recommendations provided. The quality of CPGs assessed based on according to AGREE II instrument (^H^, high; ^M^, moderate; ^L^, low). CHM, Chinese herbal medicine; TCJ, tart cherry juice; Acup, acupuncture; AT, auriculotherapy; Acupoints-based therapy includes acupuncture, acupressure, moxibustion, auricular therapy, etc.

In Figure 5, a summary of CAM recommendations for insomnia management across 17 included CPGs is presented for the benefit of clinical practitioners and researchers. In total, 22 CAM modalities were reviewed.

There were nine nutritional or natural product-related therapies, namely, valerian, chamomile, kava, hops, melissa, passiflora, tart cherry juice, melatonin, and Chinese herbal medicine (CHM). Amongst them, none of the CPGs positively endorsed the utilization of valerian, chamomile, kava, hops, melissa, passiflora, or tart cherry juice due to insufficient high-quality evidence supporting efficacy and safety. One CPG strongly opposed the use of kava not only because it had no benefit for insomnia, but there was a known risk for acute fatal liver toxicity with kava (51). One CPG published by *Chinese Sleep Research Society* recommended CHM for insomnia treatment (41), and the other two CPGs provided unclear or ambiguous recommendations of CHM (42, 53). The recommendations for melatonin varied considerably in the guidelines with some contradictory information. The use of melatonin was supported in two CPGs (41, 50), not supported in eight CPGs (42, 44, 46, 47, 51, 55–57), and presented as “*neither for nor against*” in four CPGs. Two out of eight CPGs about general insomniacs supported the use of melatonin whereas the other six did not. Melatonin was also against in two CPGs developed for ICU patients with insomnia (44) and cancer survivors with insomnia (47), respectively.

Three types of mindful movements were mentioned in the included CPGs. One CPG each recommended Tai Chi (48) and Yoga (47), respectively; no CPGs explicitly recommended or opposed the use of Qigong.

Amongst physical CAM modalities, there were no CPGs positively recommending the utlization of either massage or foot reflexology. The recommendations on acupoints-based therapy were contradictory across different CPGs. It was endorsed in one CPG (41), not supported in two CPGs (44, 56), and reported as uncertain in five CPGs.

Amongst psychological CAM modalities, neither mindfulness nor music therapy was recommended by any CPGs. The bibliotherapy, defined as the guided use of reading for therapeutic aims, was recommended in one CPG (54). Biofeedback was encouraged for insomnia treatment by one CPG (41) and was reported as “*neither for nor against*” in another three CPGs (42, 45, 57). A similar picture arose for the recommendations of light therapy.

Homeopathy and/or aromatherapy were included in four CPGs with two considering the therapies unclear/uncertain (42, 47) and two providing recommendations against their use (44, 56). One of the four CPG assessed aromatherapy targeted ICU patients with insomnia (44). The recommendation was against its use because of limited evidence of benefits for insomnia and concerns over potential respiratory irritation among ICU patients (44).

On the basis of the information provided in Figures 4, 5 and Table 1, among three high-quality and reliable CPGs (51, 53, 54), only two CAM modalities (bibliotherapy and auricular acupuncture with seed and pellet) were weakly endorsed for the treatment of chronic insomnia disorder (51). Conversely, a low-quality CPG (50) positively recommended the use of melatonin, which should be considered with caution.

The three most frequently mentioned modalities in the CPGs were, in order, melatonin, valerian, and acupoints-based therapy. Eight modalities (i.e., melissa, hops, passiflora, tart cherry juice, bibliotherapy, foot reflexology, and Qigong) were only mentioned once.

Although almost all included CPGs that did not provided definitive recommendations (or stated “neither for nor against”), they acknowledged that these CAM therapies might have potential benefits; however, the original studies underlying this evidence were methodologically poor (as noted by the authors of the meta-analyses) and thus it is difficult to reach clear and unambiguous conclusions (explicitly graded recommendations for or against the CAM use). Furthermore, none of the CPGs included recommendations to enquire about and document CAM use.

## 4. Discussion

### 4.1. Summary of findings

In the existing CPGs for insomnia treatment and/or care, CAM recommendations are distributed across five categories of CAM involving 22 therapies or products. Most recommendations are unclear, uncertain, conflicting, or “neither for nor against;” explicitly graded recommendations supporting the CAM use were scarce. Most of the included CPGs (*n* = 14, 82.4%) provided recommendations for melatonin, and of these, the negative recommendations (*n* = 8, 57.1%) were predominant. There was still considerable debate in different CPGs as to whether melatonin was recommended for use in general insomniacs. Whereas, there was no dispute that melatonin was explicitly recommended by existing CPGs not to be used in cancer survivors and ICU patients to relieve insomnia symptoms. There was some consensus (no recommendation supporting use; and recommendation against use ≥ 2) on valerian, chamomile, kava, and aromatherapy, that these modalities were recommended not to be used for insomnia treatment and care because of insufficient evidence supporting their benefits for sleep. Kava was also associated with risk for acute liver damage and death.

The reporting quality of the 17 included CPGs was moderate to high (reporting rate from 42.9 to 97.1%). Of all these CPGs, 13 were further rated as moderate to high in methodological quality. Of the 22 CAM modalities involved in the available CPGs, CHM, biofeedback, and light therapy were not bestowed any negative recommendations and were positively recommended by at least one CPG. However, the CPGs that provided such positive recommendations were rated as low quality in methodology. Of the CPGs rated as moderate to high quality overall, only bibliotherapy, Tai Chi, Yoga, and auriculotherapy were positively recommended.

It is slightly unfortunate that the aforementioned evidence could only be viewed as indirect rather than direct because the quality appraisal was performed for the entire CPG rather than the CAM section of the CPG. Hence, the development of a standardized and credible instrument to measure the quality of the CAM component of comprehensive guideline under current research topic is urgently warranted.

Taken together, the existing CPGs are generally conservative and cautious toward the application of CAM approaches for insomnia treatment and/or care.

### 4.2. Strengths, limitations, and comparison with previous systematic reviews

To the best of our current knowledge, this is the first systematic review comprehensively collecting the CAM recommendations for insomnia management from the existing CPGs as well as critically appraising the methodology and reporting quality of those CPGs. The 17 included CPGs developed by panels over a fairly diverse geographic distribution, covering North America, South America, Europe, and Asia, reflecting the diversity and representativeness of the guidelines source (Table 1). The quality of this review is further enhanced by the strong academic background of the researchers and multidisciplinary collaboration. The researchers who performed data extraction, quality assessment, and outcome analysis had backgrounds in CAM, clinical sleep science/medicine, and/or evidence-based medicine, ensuring the reliability of the current reviewed results.

Two previous systematic reviews within the same theme were published in 2016 (58) and 2021 (23), respectively. The former claimed to have included 11 CPGs which described CAM therapies, but in fact suffered from inappropriate inclusion, i.e., report/handout of treatment options for insomnia (59) were incorrectly considered as guidelines and included (58). Furthermore, this review did not evaluate the methodological and reporting quality of the included CPGs (58). The 2021 review only included six CPGs covering eight CAM modalities and did not perform reporting quality assessment (23). The 2021 review included methodological quality assessment of eligible CPGs, however such process was carried out by only two assessors and not the four recommended by the AGREE II instruction manual (23). Our review included more eligible guidelines with more CAM modalities, and adopted RIGHT checklist to appraise reporting quality of each included CPG. In addition, four trained assessors conducted the independent evaluation in the current review, and the ICC statistics showed good IRR across them. These extra inputs allowed a more comprehensive and unbiased conclusion.

Despite the strict implementation adherence to PRISMA, the current review was not without its limitations. First, the review was restricted to CPGs published in English or Chinese. Given many traditional medicine systems originate from regions of the world other than China or where English is not commonly spoken (e.g., Korea, Japan, or Iran, etc.), it is likely that there are relevant CPGs published in other languages with significant CAM recommendations that may have affected our current findings or led to different conclusions. Second, to reduce the heterogeneity across the included CPGs and enhance the applicability of our findings, only comprehensive CPG were included and assessed, and the CPG focusing on one or more specific CAM modalities were excluded. During the screening stage, at least five of the retrieved CPGs regarding traditional Chinese medicine (TCM) management for primary or secondary/comorbid insomnia were excluded (Appendix 2). It is possible that those CPGs would further enrich the results of the current review. Third, the diagnostic criteria for insomnia were only referenced from expert consensus or were not clearly described in four CPGs, which was considered to be less than rigorous and could result in potential bias. Therefore, the CAM recommendations developed in these guidelines should be interpreted/treated with caution. Finally, both AGREE II instrument and RIGHT checklist are employed to evaluate the quality of the overall CPG rather than the CAM section of each CPG. Therefore, we had to use the quality of the overall CPG to infer the quality and reliability of the CAM recommendations in each CPG. This is indirect rather than direct evidence. In order to inform health service providers with more credible CAM recommendations in insomnia management, future guidelines should incorporate broader, high-quality, and rigorous CAM evidence while ensuring methodological and reporting quality.

### 4.3. Interpretation of the current findings

The purpose of this systematic review was to identify the quantity and assess the quality of CAM recommendations in existing CPGs for insomnia management. Such information is believed to facilitate clinical practitioners, particularly those without CAM education and training experience to identify available, applicable and reliable CAM resources base therapy decisions or evidence-informed referrals upon (23, 60). In accordance with current findings, however, very few explicitly graded CAM recommendations from high-quality CPGs were identified to support communication and evidence-based decision-making between patients and their healthcare providers in the treatment and/or care of insomnia. In spite of this, CAM is utilized by approximately one-third of the Western population internationally (61) and over 50% in Asian countries such as China, the Philippines and South Korea (62). According to the NCCIH data, the three most used CAM therapies by insomnia clients, in order, are melatonin, valerian, and kava (63). In the CPGs we reviewed, the three most frequently mentioned therapies were melatonin, valerian, and acupoints-based therapy (Figure 5); kava was only mentioned in two guidelines (51, 56) and both guidelines were against its use. Three other therapies that were identified in NCCIH survey (widely used by insomnia suffers) but not included in current CPGs are relaxation-mental imagery, St. John's wort, and spiritual healing. Indeed, both therapies that are popular among insomniacs but not included in existing guidelines, and therapies that are included in CPGs but are not provided with clear (support/against) recommendations, can undermine healthcare professionals' understanding on the benefits and risks of various CAM modalities, which in turn impede their informed and shared decision-making with patients (64). These data serve as a reminder not only to clinicians of the full consideration of patient choice and preference when implementing clinical decisions based on the CPGs, but also to guideline developers of clear awareness of a potential gap between the clinical use and unambiguous guidance on clinical practice (23).

At least 10 included CPGs directly illustrated that contradictory or low-quality evidence from meta-analyses or original trials hindered the construction/generation of reliable CAM recommendations (41, 42, 44, 46–48, 51, 54, 56, 57). In addition, a previous review also indicated that the dearth of applicable CAM recommendations in the CPGs could also be explained by other factors which can affect the availability of CAM studies, including the generally negative attitudes of mainstream medical community toward CAM therapies, and a lack of CAM funding (23). Regardless of the reason, the dearth of applicable and reliable CAM recommendations in existing guidelines is a concern. This is likely to result in the underuse of beneficial CAM therapies, and/or the continued utilization of potentially harmful CAM therapies (23). The latter is obviously of greater concern as it links to the safety challenges in clinical practice, particularly with regard to drug-related liver injury (65). After all, over-the-counter natural products (including herbs) have been the “mainstay” of CAM options in insomnia treatment, yet rarely have to undergo the multiple preclinical and phased clinical trials before approval of marketing and use, which is the case for modern pharmaceuticals (65). Of the 22 CAM modalities covered in this review, seven were phytotherapeutics (i.e., valerian, chamomile, kava, hops, melissa, passiflora, and CHM; Figure 5). Whereas, by analyzing the data of drug-induced liver injury (DILI) in South Korea, Suk et al. reported that herbal medications and dietary supplements were found to cause DILI in 27.5 and 13.7%, respectively (66). In Japan, 10 and 7% of DILI were reported to be caused by proprietary herbal dietary supplements and CHM, respectively (67). Given the widespread use of CHM nationwide, China provides more reliable data based on a larger sample in the investigation of liver damage caused by CHM. In two systematic analyses consisting of 9,335 and 24,112 patients with DILI respectively, CHM was found responsible for DILI in 18.6 and 21.2% cases (68). Similarly, CAM therapies are commonly used by consumers in conjunction with orthodox medical treatments (69). A report released by Australian National Prescribing Service indicated that half of all CAM users surveyed acknowledged using CAM modalities (e.g., herbs, vitamins and mineral supplements, other nutritional supplements, etc.) on the same day as taking on the same day as taking prescription or non-prescription medicines (70). This is of concern given the growing evidence of potential and confirmed adverse effects associated with possible CAM–prescribed drug interactions or compounding of effects (69).

Regrettably, none of the reviewed CPGs included recommendations enquiring about and/or documenting CAM use. This represents a major missed opportunity to invite patients to participate in shared decision-making about appropriate use of CAM, equip doctors and nurses with knowledge about CAM and evidence for them, and to provide person-centered care where there is an illustrated benefit (27).

During the literature screening process, one CPG (71) focusing on the treatment of menopausal sleep disorders was excluded due to a lack of clear description of the systems/methods used for grading the evidence and recommendations (Appendix 2). None of the 17 included CPGs in this review addressed the same topic. Because of the intolerance to the adverse events of conventional pharmacotherapy (i.e., hormonal replacement therapy and/or psychotropic substances), up to 50% of peri- and post- menopausal women worldwide seek assistance from CAM therapies, including acupuncture, massage, yoga, herbalism, and dietotherapy for symptomatic relief (72). Likewise, roughly two-thirds of pregnant woman report poor sleep, and those with insomnia usually turn to non-pharmacological and natural CAM modalities due to lack of confidence in medications (73). None of the included CPGs addressed those two specific populations. Therefore, there is still a need for guidelines that include quality CAM advice for the management of insomnia during these two special stages of women's life. Conversely, many recommendations of the CPGs targeting insomnia were general rather than specific populations and could overlap (Table 1). It is hence worth considering integrating the efforts, expertise, and resources of multiple organizations via international collaboration as a pathway to support the development of high-quality international CPG while reduce the number of redundant CPGs (74).

### 4.4. Implications for CPG development/updates and CAM clinical practice

#### 4.4.1. Implications for CPG development/updates

The CPGs aim to bridge the gap between research evidence and clinical practice and should thereby be developed using the most rigorous methodology (74). The trustworthy CPGs can lend to widespread use of efficient medical practices among clinicians and have more potential to improve patient outcomes and satisfaction (75, 76). In addition, they could help modify the behavior of clinicians (38) and be used as a good tool to respond to public health issues (38, 77). Although the overall quality of the included CPGs is acceptable, there is still plenty of room for improvement (Figure 4). Three reviewed CPGs were poor in quality and comprehensiveness. Adoption of such CPGs is often associated with ineffective treatment of patients or even endangering their health (76), difficulties with standardization of care, adaptation, and implementation in resource-limited settings (78), wrong direction of clinical research, and even waste of medical resources (38). Hence, in future updates, those CPGs achieved lower scores in individual or overall domain(s) should be optimized according to specifics in the AGREE II instrument (36) and RIGHT checklist (39), or other available resources (e.g., CPG-related principles, frameworks, and criteria, etc.) (23). Based on the findings in this review, the “applicability” domain of AGREE II instrument usually failed to be scored satisfactorily. Many other studies have reported similar findings (74). Low applicability of the CPG can reduce its rate of use in daily practice, hinder the maximization of its positive impact on healthcare (74), and/or its clinical generalization (38). A review targeting physician adherence to CPGs indicated that as many as 38% of physicians considered CPGs as inconvenient or too difficult to utilize (79). The “rationale/explanation for recommendations” domain of RIGHT checklist was generally scored as unsatisfactory as well. Correspondingly, for future insomnia CPGs, its application attribute (i.e., facilitators and barriers to CPG's application, advice, tools, and potential resource implications on transferring the recommendations into practice, etc.) and basis of recommendations (i.e., values and preferences of the target population, equity, feasibility, and acceptability) in particular deserve more attention.

A critical research gap of concern is a lack of established instrumentation that can be used to assess the CAM sections within the comprehensive CPGs. Furthermore, whilst specialized CAM CPGs were not included in this review, the existing tools [AGREE II instrument (36) and RIGHT checklist (39)] appear not to be applicable to the quality appraisal of such CPGs. Take TCM-specialized CPGs for instance, it is crucial to assess (1) whether the recommendations based on TCM syndrome patterns, and whether the patterns included in the CPG is comprehensive; (2) besides the recommendations of CHM, whether the recommendations cover the combined use of CHM and hypnotics/sedatives; (3) whether the recommendations clarify which modality must be performed by medical personnel and which modality can be self-administered by the patient (e.g., acupuncture must be delivered by acupuncturist, while auricular acupressure might be self-administered by the patient, although both modalities are acupoints-based). None of these elements can be assessed using the existing CPG appraisal tool. Hence, there is a strong need to carefully develop two practical, valid, and reliable instrumentations for assessing the quality of CAM recommendations in comprehensive CPGs and assessing the quality of specialized CAM CPGs, respectively. As suggested, dimensions such as validity, clinical applicability, clinical flexibility, clarity, reliability/reproducibility and multidisciplinary process might be taken into account when developing such tools (80).

We are also aware that most of the included CPGs were developed by the medical societies/associations (41, 42, 44–46, 53, 55–57). A previous study concluded that CPGs published by medical societies were often limited in quality (74). This could be due to medical societies/associations having a less diverse development panel consisting of members beyond physicians. The perspective of other healthcare professionals and community members is required to improve not only the quality of some domains within a CPG, but also the implementability (74). For CPGs that comprise a CAM component (particularly when developing recommendations for CAMs with significant cultural/geographical/religious attributes), it is even more crucial to establish a multidisciplinary development panel (i.e., epidemiologists, clinicians/registered nurse, CAM practitioners with specialized expertise, methodologists, health economist, and consumers) rather than a mainstream medicine physician-only panel. Such stakeholder engagement, particularly with diverse groups of end-users, can allow for an evidence-based, transparent, and systematic approach to create a CPG that is relevant and fit for purpose (27). This has not been given the attention it deserves. Reviews from the UK and Germany have revealed that only 10–25% of the CPGs consider CAM modalities in their recommendations (27, 81) and development team rarely seek contributions from CAM specialists (27, 28). This condition is also corroborated in our current review. In a UK study, 223 CAM organizations were sent questionnaires to answer, “Which complementary and alternative therapies benefit which conditions?” According to the results, the top six therapies advocated and highly provided by professional CAM practitioners for the treatment of insomnia were aromatherapy, hypnotherapy, massage, reflexology, reiki, and yoga (82). However, melatonin, valerian, and acupoints-based therapy, which are most frequently mentioned in the reviewed CPGs, were not in this list; meanwhile, hypnotherapy and reiki in the list were not even included in any existing CPG. This again confirms the gap between patient choice in therapies and the provision of professional guidelines for these therapies from clinicians.

#### 4.4.2. Implications for CAM clinical practice

As a previous study highlighted, the quality evaluation scores of a CPG could not represent how it had affected clinical practice in the years following its publication (38). Also, those so-called “recommended with modification” and “not recommended” classification for guidelines only referred to the deficiencies in their reporting information and development process, but should not be equated exclusively with the fact that the therapies covered by these guidelines are of no clinical practice value (38). Briefly, the clinical value of any CAM therapy should not be simply affirmed or repudiated if the CPG only rely on low quality evidence or input from limited professions (38). Instead, it should be determined in an objective and comprehensive review of the adequate and solid evidence. In summary, we suggest that clinicians place a higher priority to CAM recommendations provided by high-quality CPGs in combination with specific clinical settings and suitable patient population. CAM recommendations in low-quality CPGs should not however be repudiated outright but should be withheld for the time being and determined once more high-quality evidence is accumulated. In addition, healthcare service providers should pay attention to the timeliness of CPGs in any case (38), although keeping CPGs updated and reflective of the sheer volume of the latest evidence is indeed a challenge due to the time-consuming, labor-intensive, and expensive process (27). After all, the failure to include new evidence might result in inability to translate evidence into health outcomes in a timely manner (27). However, the requirement for incorporation of the latest evidence on CAM is even greater given the rapidly expanding evidence base (27).

Of course, it is undeniable that the current evidence of the effectiveness and safety associated with CAM is mixed, with some modalities remaining controversial (17, 61). The general knowledge gap makes many mental health practitioners uncomfortable when discussing CAM therapies and therefore is likely to prevent them from communicating or extending evidence-based CAM advice to patients (61). More worryingly, evidence suggests that the rate of non-disclosure of CAM use is high (27). For a variety of reasons (e.g., their perceptions that the medical physicians lack relevant knowledge, fear of being admonished or evoking negative responses, etc.), a considerable proportion of CAM users self-prescribed, rely on advice from friends and family to guide their CAM decisions, and/or did not inform or discuss with physicians about their CAM use (61). These irrational utilizations contribute to many of the associated medical risks, such as drug-herb interaction or side effects of the herbs (17, 61). In contrast, initiating dialogue about CAM use during the medical encounter is helpful to minimize risk and to forge a better therapeutic alliance, and thereupon improve the patient-provider relationship and patient's satisfaction (83). For CAM which is outside orthodox medicine and with less understanding, CPGs are used by mainstream healthcare professionals to inform their practice decisions (23). Briefly, the CPG may be a linchpin to trigger the dialogue/discussion regarding CAM between these professionals and their customers (insomnia suffers). Considering the popularity of CAM use in general population worldwide (with use prevalence ranged from 9.8 to 76%) (84), it is essential for future CPGs to include more high-quality and definitive CAM recommendations to help initiate such dialogue in the clinical settings.

Whilst existing CPGs have provided recommendations for 22 CAM therapies, there were still some other modalities which also showed potential in insomnia management that have not been reported. These modalities included but were not limited to pharmacological/non-pharmacological approaches in Ayurveda (e.g., Vishnukranta, Insomrid Tablet, and Shirodhara, etc.) (85), spiritual and religious interventions (e.g., prayer, religious meditation, and spiritual connection techniques, etc.) (86), hypnotherapy and hypnotherapy-like treatments (e.g., hypnotherapy, autogenic training, and guided hypnosis-like imagery, etc.) (87). Collecting evidence and identifying its quality for these therapies therefore may be considered in future research, and recommendations should be provided accordingly to serve as the basis for further updates of the existing CPGs.

## 5. Conclusions

Despite the popularity of CAM use in insomnia management, existing CPGs were conservative and cautious in recommending the utilization of these therapies. The lack of adequate high-quality clinical evidence and a lack of a multidisciplinary development panel possibly underlie this position. The only consensus was that valerian, chamomile, kava, and aromatherapy were not recommended for the treatment and care of insomnia because of their proven risks and/or very limited benefits. To avoid the continued utilization of potentially harmful CAM modalities, and/or the underuse of beneficial CAM modalities, performing more stringently designed trials that can produce high-quality evidence and thus facilitate CPGs to develop clear (pro or con) recommendations for specific CAM therapy are required. Engaging a range of stakeholders including clinicians, CAM practitioners, epidemiologists, methodologists, health economist, consumers, etc. in future updates of CPGs are also warranted. The lack of comprehensive recommendations for healthcare service providers to enquire about CAM use by their customers represents a great missed opportunity for shared decision-making. Therefore, inclusion of recommendations to enquire about and document CAM use in future updates/new development of CPGs is also suggested. In addition, the development of a measurement specifically applicable to evaluate the quality of CAM recommendations in comprehensive CPGs is urgently needed. It is also required to be used in combined with AGREE II instrument and RIGHT checklist as a pathway to improve the overall quality of comprehensive CPGs that contain a CAM section.

## Data availability statement

The original contributions presented in the study are included in the article/Supplementary material, further inquiries can be directed to the corresponding authors.

## Author contributions

F-YZ: conceptualization, investigation, methodology, formal analysis, data curation, literature quality assessment, and writing—original draft. PX: conceptualization, investigation, methodology, and data curation. GK: conceptualization and writing—review and editing. RC and ZZ: writing—review and editing and project administration. W-JZ and Y-MW: investigation and literature quality assessment. Q-QF: investigation, methodology, validation, formal analysis, data curation, and data visualization. All authors contributed to the article and approved the submitted version.
