# Supplementary material for: Identifying complementary and alternative medicine recommendations for insomnia treatment and care: a systematic review and critical assessment of comprehensive clinical practice guidelines
Source: Front Public Health. 2023 Jun 15;11:1157419. doi: 10.3389/fpubh.2023.1157419 (PMC10308125; doi:10.3389/fpubh.2023.1157419)
Supplement: Supplementary file 1 [file Data_Sheet_1.pdf]

Appendices

Appendix 1 Search strategy for each database

MEDLINE (via PubMed)	#1 clinical practice guideline[Title/Abstract]<br>#2 clinical guideline[Title/Abstract]<br>#3 practice guideline[Title/Abstract]<br>#4 guideline[Title/Abstract]<br>#5 guidance[Title/Abstract]<br>#6 recommendation[Title/Abstract]<br>#7 consensus[Title/Abstract]<br>#8 experts consensus[Title/Abstract]<br>#9 statement[Title/Abstract]<br>#10 #1 or #2 or #3 or #4 or #5 or #6 or #7 or #8 or #9<br>#11 insomnia[Title/Abstract]<br>#12 sleep initiation and maintenance disorders[Title/Abstract]<br>#13 sleep disorder[Title/Abstract]<br>#14 dyssomnias[Title/Abstract]<br>#15 wakefulness[Title/Abstract]<br>#16 sleep Deprivation[Title/Abstract]<br>#17 awake[Title/Abstract]<br>#18 wake[Title/Abstract]<br>#19 #11 or #12 or #13 or #14 or #15 or #16 or #17 or #18<br>#20 #10 and #19
EMBASE (via OVID)	#1 'clinical practice guideline':ab,ti<br>#2 'clinical guideline':ab,ti<br>#3 'practice guideline':ab,ti<br>#4 'guideline':ab,ti<br>#5 'guidance':ab,ti<br>#6 'recommendation':ab,ti<br>#7 'consensus':ab,ti<br>#8 'experts consensus':ab,ti<br>#9 'statement':ab,ti<br>#10 'clinical practice guideline':ab,ti OR 'clinical guideline':ab,ti OR 'practice guideline':ab,ti OR 'guideline':ab,ti OR 'guidance':ab,ti OR 'recommendation':ab,ti OR 'consensus':ab,ti OR 'experts consensus':ab,ti OR 'statement':ab,ti<br>#11 'insomnia':ab,ti<br>#12 'sleep initiation and maintenance disorders':ab,ti<br>#13 'sleep disorder':ab,ti<br>#14 'dyssomnias':ab,ti<br>#15 'wakefulness':ab,ti<br>#16 'sleep deprivation':ab,ti<br>#17 'awake':ab,ti

	#18 'wake':ab,ti<br>#19 'insomnia':ab,ti OR 'sleep initiation and maintenance disorders':ab,ti OR 'sleep disorder':ab,ti OR 'dyssomnias':ab,ti OR 'wakefulness':ab,ti OR 'sleep deprivation':ab,ti OR 'awake':ab,ti OR 'wake':ab,ti<br>#20 ('clinical practice guideline':ab,ti OR 'clinical guideline':ab,ti OR 'practice guideline':ab,ti OR 'guideline':ab,ti OR 'guidance':ab,ti OR 'recommendation':ab,ti OR 'consensus':ab,ti OR 'experts consensus':ab,ti OR 'statement':ab,ti) AND ('insomnia':ab,ti OR 'sleep initiation and maintenance disorders':ab,ti OR 'sleep disorder':ab,ti OR 'dyssomnias':ab,ti OR 'wakefulness':ab,ti OR 'sleep deprivation':ab,ti OR 'awake':ab,ti OR 'wake':ab,ti)
AMED: Allied and Complementary Medicine Database	#1 AB clinical practice guideline OR TI clinical practice guideline<br>#2 AB clinical guideline OR TI clinical guideline<br>#3 AB practice guideline OR TI practice guideline<br>#4 AB guideline OR TI guideline<br>#5 AB guidance OR TI guidance<br>#6 AB recommendation OR TI recommendation<br>#7 AB consensus OR TI consensus<br>#8 AB experts consensus OR TI experts consensus<br>#9 AB statement OR TI statement<br>#10 #1 OR #2 OR #3 OR #4 OR #5 OR #6 OR #7 OR #8 OR #9<br>#11 AB insomnia OR TI insomnia<br>#12 AB (sleep initiation and maintenance disorders) OR TI (sleep initiation and maintenance disorders)<br>#13 AB sleep disorder OR TI sleep disorder<br>#14 AB dyssomnias OR TI dyssomnias<br>#15 AB awake OR TI awake<br>#16 AB wake OR TI wake<br>#17 #11 OR #12 OR #13 OR #14 OR #15 OR #16<br>#18 (#11 OR #12 OR #13 OR #14 OR #15 OR#16) AND (#10 AND #17)
China National Knowledge Infrastructure (CNKI)	(篇关摘=临床实践指南) OR (篇关摘=临床指南) OR (篇关摘=指南) OR (篇关摘=共识) AND (篇关摘=失眠) OR (篇关摘=失眠症) OR (篇关摘=原发性失眠) OR (篇关摘=睡眠障碍) OR (篇关摘=睡眠-觉醒障碍) OR (篇关摘=睡眠) OR (篇关摘=不寐)
Wanfang database	题名或关键词:(临床实践指南 or 临床指南 or 指南 or 共识) and 题名或关键词:(失眠 or 失眠症 or 原发性失眠 or 睡眠障碍 or 睡眠-觉醒障碍 or 睡眠 or 不寐)
Chongqing VIP database (CQVIP)	题名或关键词=临床实践指南 OR 题名或关键词=临床指南 OR 题名或关键词=指南 OR 题名或关键词=共识 AND 题名或关键词=失眠 OR 题名或关键词=失眠症 OR 题名或关键词=原发性失眠 OR 题名或关键词=睡眠障碍 OR 题名或关键词=睡眠-觉性障碍 OR 题名或关键词=睡眠 OR 题名或关键词=不寐
China biomedical literature service system (SinoMed)	"临床实践指南"[标题:智能] OR "临床指南"[标题:智能] OR "指南"[标题:智能] OR "共识"[标题:智能] AND "失眠"[标题:智能] OR "失眠症"[标题:智能] OR "原发性失眠"[标题:智能] OR "睡眠障碍"[标题:智能] OR "睡眠-觉性障碍"[标题:智能] OR "睡眠"[标题:智能] OR "不寐"[标题:智能]
NCCIH website	https://www.nccih.nih.gov/health/providers/clinicalpractice
Guideline related websites	Guidelines International Network (https://g-i-n.net/)
	National Institute for Health and Clinical Excellence (https://www.nice.org.uk/)
	British Columbia guideline (http://www.bcguidelines.ca/alphabetica)
	National Guideline Clearinghouse (https://www.ahrq.gov/gam/index.html)
	Scottish Intercollegiate Guidelines Network (https://www.sign.ac.uk/)
	Canadian Medical Association: Clinical Practice Guideline (https://joulecma.ca/)

Appendix 2 Excluded clinical practice guidelines with detailed reasons of irrelevance

References	Reasons for exclusion
Ashton H. Guidelines for the rational use of benzodiazepines. When and what to use. *Drugs*, 1994; 48(1):25-40. doi: 10.2165/00003495-199448010-00004.	without CAM information
Bjerre LM, Farrell B, Hogel M, Graham L, Lemay G, McCarthy L, Raman-Wilms L, Rojas-Fernandez C, Sinha S, Thompson W, Welch V, Wiens A. Deprescribing antipsychotics for behavioural and psychological symptoms of dementia and insomnia: Evidence-based clinical practice guideline. *Can Fam Physician*, 2018; 64(1):17-27.	without CAM information
Chesson AL Jr, Anderson WM, Littner M, Davila D, Hartse K, Johnson S, Wise M, Rafecas J. Practice parameters for the nonpharmacologic treatment of chronic insomnia. An American Academy of Sleep Medicine report. Standards of Practice Committee of the American Academy of Sleep Medicine. *Sleep*, 1999; 22(8):1128-1133.	earlier version of the included CPG (45)
China Association of Acupuncture-Moxibustion. Evidence-based guideline of clinical practice with acupuncture and moxinustion [article in Chinese]. China Press of Tradition Chinese Medicine: 2014.	non-comprehensive CPGs (TCM specialized)
China Academy of Chinese Medical Scinences. Evidence-based clinical practice guidelines of traditional Chinese medicine: Internal medicine of traditional Chinese medicine [article in Chinese]. China Press of Tradition Chinese Medicine: 2011.	non-comprehensive CPGs (TCM specialized)
China Academy of Chinese Medical Sciences. Chinese medicine clinical practice guidelines for insomnia (WHO/WPO) [article in Chinese]. *World J Sleep Med*, 2016; 3(1):8-25.	non-comprehensive CPGs (TCM specialized)
Gupta R, Das S, Gujar K, Mishra KK, Gaur N, Majid A. Clinical Practice Guidelines for Sleep Disorders. *Indian J Psychiatry*, 2017; 59(Suppl 1):S116-S138.	without clear description of the systems or methods used for grading the evidence and recommendations
Koch AK, Langhorst J. Phytotherapie in der S3-Leitlinie, Nicht erholsamer Schlaf/Schlafstörungen. *Zeitschrift für Phytotherapie*, 2018; 39(6): 257-259.	not published in English or Chinese
Lam WC, Zhong L, Liu Y, Shi N, Ng B, Ziea E, Bian Z, Lu A. Hong Kong Chinese Medicine Clinical Practice Guideline for Cancer Palliative Care: Pain, Constipation, and Insomnia. *Evid Based Complement Alternat Med*, 2019; 2019:1038206. doi: 10.1155/2019/1038206.	non-comprehensive CPGs (TCM specialized)
Malan L, Dlamini N. Clinical practice guidelines for insomnia disorder. *South African Family Practice*, 2017; 59(3): 45-51.	without CAM information
Morgenthaler T, Kramer M, Alessi C, Friedman L, Boehlecke B, Brown T, Coleman J, Kapur V, Lee-Chiong T, Owens J, Pancer J, Swick T; American Academy of Sleep Medicine. Practice parameters for the psychological and behavioral treatment of insomnia: an update. An American Academy of Sleep Medicine report. *Sleep*, 2006; 29(11):1415-1419.	earlier version of the included CPG (45)
Pamula Y, Nixon GM, Edwards E, Teng A, Verginis N, Davey MJ, Waters K, Suresh S, Twiss J, Tai A. Australasian Sleep Association clinical practice guidelines for performing sleep studies in children. *Sleep Med*, 2017; 36 Suppl 1:S23-S42.	without adults information
Pottie K, Thompson W, Davies S, Grenier J, Sadowski CA, Welch V, Holbrook A, Boyd C, Swenson R, Ma A, Farrell B. Deprescribing benzodiazepine receptor agonists:	without CAM information

Evidence-based clinical practice guideline. *Can Fam Physician*, 2018; 64(5):339-351.	
Praharaj SK, Gupta R, Gaur N. Clinical practice guideline on management of sleep disorders in the elderly. *Indian J Psychiatry*, 2018; 60(Suppl 3):S383-S396.	without CAM information (for insomnia)
Riemann D, Fischer J, Mayer G, Peter HJ. The guidelines for 'non-restorative sleep': relevance for the diagnosis and therapy of insomnia. *Somnologie-Schlafforschung und Schlafmedizin*, 2003; 7(2): 66-76.	without clear description of the systems or methods used for grading the evidence and recommendations
Silvestri R, Aricò I, Bonanni E, Bonsignore M, Caretto M, Caruso D, Di Perri MC, Galletta S, Lecca RM, Lombardi C, Maestri M, Miccoli M, Palagini L, Provini F, Puligheddu M, Savarese M, Spaggiari MC, Simoncini T. Italian Association of Sleep Medicine (AIMS) position statement and guideline on the treatment of menopausal sleep disorders. *Maturitas*, 2019; 129:30-39.	without clear description of the systems or methods used for grading the evidence and recommendations
Smyth A, Jenkins M, Dunham M, Kutzer Y, Taheri S, Whitehead L. Systematic review of clinical practice guidelines to identify recommendations for sleep in type 2 diabetes mellitus management. *Diabetes Res Clin Pract*, 2020; 170:108532.	without CAM information
Sparks A, Cohen A, Adekola L, Adjao S, Balderson B, Rachel Berger, Chau J, DeWitt C, Kavanagh M, Mayer C, Scearce T, Shyn S, Slomovits G, Stedronsky A, Sturgis M, Wang J. Insomnia guideline. https://wa.kaiserpermanente.org/static/pdf/public/guidelines/insomnia.pdf	without clear description of the systems or methods used for grading the evidence and recommendations
Taillard J. Procédure de réalisation des Tests de Maintien d'Eveil et valeurs normatives. https://www.sfrms-sommeil.org/wp-content/uploads/2012/10/sfrms_recoR2bis.pdf	not published in English or Chinese
Wang WD, Li T, Yan X, Lu XY. Individual-based Chinese medicine clinical practice guidelines for insomnia [article in Chinese]. *World J Sleep Med*, 2016, 3(2):65-79.	non-comprehensive CPGs (TCM specialized)
Williams Buckley A, Hirtz D, Oskoui M, Armstrong MJ, Batra A, Bridgemohan C, Coury D, Dawson G, Donley D, Findling RL, Gaughan T, Gloss D, Gronseth G, Kessler R, Merillat S, Michelson D, Owens J, Pringsheim T, Sikich L, Stahmer A, Thurm A, Tuchman R, Warren Z, Wetherby A, Wiznitzer M, Ashwal S. Practice guideline: Treatment for insomnia and disrupted sleep behavior in children and adolescents with autism spectrum disorder: Report of the guideline development, dissemination, and implementation subcommittee of the American Academy of Neurology. *Neurology*, 2020; 94(9):392-404.	without adults information
Zhang P, Zhao ZX. Interpretation of the Chinese guidelines for the diagnosis and treatment of insomnia in adults [article in Chinese]. *Chinese Journal of Modern Neurological Disorders*, 2013; 13(05):363-367.	earlier version of the included CPG (42)

Abbreviations CPG(s), clinical practice guideline(s); CAM, complementary and alternative medicine; TCM, Traditional Chinese Medicine.

Appendix 3 Inter-rater reliability for each domain of the AGREE II instrument

Domains	ICCs	95% *CI*		*p*
		Lower-bound	Upper-bound	
Scope and purpose	0.74	0.56	0.88	< 0.01
Stakeholder involvement	0.75	0.57	0.89	< 0.01
Rigor of development	0.89	0.79	0.95	< 0.01
Clarity of presentation	0.73	0.54	0.88	< 0.01
Applicability	0.82	0.66	0.92	< 0.01
Editorial independence	0.90	0.81	0.96	< 0.01
Overall	0.85	0.71	0.93	< 0.01

Abbreviations: ICCs, Intraclass Correlation Coefficients; CI, confidence intervals.

Appendix 4 Methodological quality of each clinical practice guideline appraised by the AGREE II instrument

Author, year	Six domains of AGREE II						Overall score	Overall quality
	Scope and purpose (%)	Stakeholder involvement (%)	Rigor of development (%)	Clarity of presentation (%)	Applicability (%)	Editorial independence (%)		
Artiach *et al*. 2009 (54)	90.3	73.6	75.5	84.7	37.5	58.3	70.0	H
Baker *et al*. 2014 (48)	68.1	47.2	63.5	81.9	17.7	39.6	53.0	M
Bloom *et al*. 2009 (49)	75.0	45.8	50.5	68.1	27.1	60.4	54.5	M
Choi *et al*. 2020 (55)	73.6	63.9	65.6	73.6	39.6	62.5	63.1	M
Denlinger *et al*. 2009 (43)	88.9	66.7	24.5	45.8	18.8	43.8	48.1	L
Devlin *et al*. 2018 (44)	65.3	63.9	66.1	81.9	33.3	85.4	66.0	M
Edinger *et al*. 2021 (45)	79.2	59.7	65.1	83.3	30.2	72.9	65.1	M
Han *et al*. 2017 (41)	70.8	48.6	41.7	80.6	56.3	0	49.7	L
Howell *et al*. 2013 (47)	80.6	65.3	68.8	62.5	40.6	83.3	66.9	M
Leopando *et al*. 2003 (50)	51.4	31.9	46.4	69.4	16.7	0	36.0	L
Mysliwiec *et al*. 2020 (51)	86.1	76.4	74.5	76.4	57.3	77.1	74.6	H
Pinto *et al*. 2010 (52)	54.2	34.7	20.3	48.6	26.0	0	30.6	L
Qaseem *et al*. 2016 (53)	88.9	69.4	77.1	84.7	30.2	89.6	73.3	H
Riemann *et al*. 2017 (56)	80.6	52.8	65.6	61.1	29.2	87.5	62.8	M
Sateia *et al*. 2017 (46)	76.4	69.4	71.4	81.9	47.9	56.3	67.2	M
Schutte-Rodin *et al*. 2008 (57)	72.2	51.4	49.5	70.8	33.3	60.4	56.3	M
Zhang *et al*. 2018 (42)	73.6	68.1	19.3	83.3	32.3	41.7	53.1	M
Mean ± SD	75.0 ± 11.1	58.2 ± 13.2	55.6 ± 19.3	72.9 ± 12.3	33.8 ± 11.9	54.0 ± 30.1	58.3 ± 12.4	

Notes low quality, overall scores < 50%; moderate quality, 50% ≤ overall scores ≤ 70%; high quality, overall scores > 70%.

Abbreviations L, low quality; M, moderate quality; H, high quality.

Appendix 5 Reporting quality of each clinical practice guideline appraised by the RIGHT checklist

	Artiach *et al.* 2009 (54)	Baker *et al.* 2014 (48)	Bloom *et al.* 2009 (49)	Choi *et al.* 2020 (55)	Denlinger *et al.* 2009 (43)	Devlin *et al.* 2018 (44)	Edinger *et al.* 2021 (45)	Han *et al.* 2017 (41)	Howell *et al.* 2013 (47)	Leopando *et al.* 2003 (50)	Mysliwiec *et al.* 2020 (51)	Pinto *et al.* 2010 (52)	Qaseem *et al.* 2016 (53)	Riemann *et al.* 2017 (56)	Sateia *et al.* 2017 (46)	Schutte-Rodin *et al.* 2008 (57)	Zhang *et al.* 2018 (42)
Domain 1: Basic information																	
Title/subtitle																	
1a	Y	Y	Y	Y	Y	Y	Y	Y	Y	Y	Y	Y	Y	Y	Y	Y	Y
1b	N	N	N	N	Y	N	N	N	N	N	Y	N	N	N	N	N	Y
1c	Y	Y	Y	Y	Y	Y	Y	Y	Y	Y	Y	Y	Y	Y	Y	Y	Y
Executive summary																	
2	Y	N	Y	Y	Y	N	Y	N	Y	Y	Y	N	Y	Y	Y	Y	N
Abbreviations and acronyms																	
3	Y	Y	Y	Y	Y	N	Y	Y	Y	Y	Y	Y	Y	Y	Y	Y	Y
Corresponding developer																	
4	N	N	Y	Y	Y	Y	Y	Y	Y	Y	Y	Y	Y	Y	Y	Y	Y
Domain 2: Background																	
Brief description of the health problem(s)																	
5	Y	N	Y	Y	Y	N	Y	Y	Y	Y	Y	Y	Y	Y	Y	Y	Y
Aim(s) of the guideline																	

and specific objectives																	
6	Y	Y	Y	Y	Y	Y	Y	Y	Y	Y	Y	Y	Y	Y	Y	Y	Y
Target populations																	
7a	Y	Y	Y	Y	Y	Y	Y	Y	Y	Y	Y	Y	Y	Y	Y	Y	Y
7b	Y	N	N	N	N	N	N	Y	N	N	Y	Y	Y	Y	Y	N	Y
End users and settings																	
8a	Y	Y	Y	Y	Y	Y	Y	Y	Y	Y	Y	N	Y	Y	Y	Y	Y
8b	Y	Y	Y	Y	N	N	N	N	N	Y	Y	N	Y	N	N	N	N
Guideline development groups																	
9a	Y	N	Y	Y	Y	Y	N	Y	N	N	Y	N	Y	Y	Y	Y	N
9b	Y	N	Y	Y	Y	Y	Y	Y	Y	N	N	N	Y	Y	N	Y	Y
Domain 3: Evidence																	
Health care questions																	
10a	Y	Y	Y	Y	N	Y	Y	Y	Y	N	Y	N	Y	Y	Y	Y	N
10b	N	N	N	N	N	Y	N	N	N	N	Y	N	Y	N	Y	N	N
Systematic reviews																	
11a	Y	Y	Y	Y	N	Y	Y	Y	Y	N	Y	Y	Y	Y	Y	Y	N
11b	Y	Y	Y	Y	N	Y	Y	Y	Y	N	Y	N	Y	Y	Y	Y	N
Assessment of the certainty of the body of evidence																	
12	Y	Y	Y	Y	Y	Y	N	Y	N	Y	Y	Y	Y	Y	Y	Y	Y
Domain 4: Recommendations																	

Recommendations																	
13a	Y	Y	Y	Y	Y	Y	Y	Y	Y	Y	Y	Y	Y	Y	Y	Y	Y
13b	Y	Y	Y	Y	Y	Y	N	Y	N	Y	Y	Y	Y	Y	Y	Y	Y
13c	Y	Y	Y	Y	Y	Y	N	Y	N	y	Y	Y	Y	Y	Y	Y	Y
Rationale/explanation for recommendations																	
14a	N	N	N	N	N	Y	N	N	N	N	Y	N	Y	N	Y	N	N
14b	Y	N	N	N	N	Y	N	N	N	N	Y	N	Y	N	Y	N	N
14c	N	N	N	N	N	N	N	N	N	N	Y	Y	N	N	Y	N	N
Evidence to decision processes																	
15	Y	N	Y	Y	N	Y	N	Y	Y	Y	Y	N	Y	Y	Y	Y	Y
Domain 5: Review and quality assurance																	
External review																	
16	Y	Y	N	Y	N	N	N	N	Y	N	Y	N	Y	N	N	N	N
Quality assurance																	
17	Y	Y	Y	Y	N	Y	N	N	Y	Y	Y	N	Y	Y	Y	Y	N
Domain 6: Funding, declaration and management of interests																	
Funding source(s) and role(s) of the funder																	
18a	Y	Y	Y	Y	N	Y	Y	N	Y	N	Y	N	Y	Y	Y	N	N

18b	N	N	Y	N	N	Y	Y	N	Y	N	Y	N	N	Y	Y	N	N
Declaration and management of interest																	
19a	Y	Y	Y	Y	Y	Y	N	N	Y	N	Y	N	Y	Y	Y	Y	N
19b	Y	N	Y	Y	Y	Y	N	N	Y	N	Y	N	Y	Y	Y	Y	N
Domain 7: Other information																	
Access																	
20	Y	Y	Y	Y	Y	Y	Y	Y	Y	Y	Y	Y	Y	Y	Y	Y	Y
Suggestions for further research																	
21	Y	N	Y	Y	N	Y	Y	N	Y	N	Y	N	Y	Y	Y	Y	N
Limitations of the guideline																	
22	N	N	N	Y	N	Y	N	N	Y	N	Y	N	Y	N	Y	N	N
No. of reported items (%)	28 (80.0)	19 (54.3)	27 (77.1)	28 (80.0)	19 (54.3)	27 (77.1)	18 (51.4)	20 (57.1)	24 (68.6)	17 (48.6)	34 (97.1)	15 (42.9)	32 (91.4)	27 (77.1)	31 (88.6)	24 (68.6)	17 (48.6)

Notes Explanation for each item can refer to the link http://www.right-statement.org/right-statement/checklist
